# Missense Mutant p53 Transactivates Wnt/β-Catenin Signaling in Neighboring p53-Destabilized Cells through the COX-2/PGE2 Pathway

**DOI:** 10.1158/2767-9764.CRC-24-0471

**Published:** 2025-01-03

**Authors:** Mizuho Nakayama, Hiroshi Saito, Kazuhiro Murakami, Hiroko Oshima, Masanobu Oshima

**Affiliations:** 1 Division of Genetics, Cancer Research Institute, Kanazawa University, Kanazawa, Japan.; 2 WPI Nano-Life Science Institute (NanoLSI), Kanazawa University, Kanazawa, Japan.; 3 Department of Gastrointestinal Surgery, Kanazawa University, Kanazawa, Japan.; 4 Division of Epithelial Stem Cell Biology, Cancer Research Institute, Kanazawa University, Kanazawa, Japan.

## Abstract

Missense-type p53 mutations have shown to acquire novel oncogenic roles through a gain-of-function mechanism. However, there is intratumor heterogeneity in stabilization of mutant p53 protein, and it has not been well understood about the interaction between p53-stabilized and -destabilized cells in the same tumors. We established mouse intestinal tumor–derived organoids carrying *Apc*^Δ716^, *Kras*^G12D^, and *Tgfbr2*^−/−^ mutations with *Trp53*^R270H^ or *Trp53*^Null^ mutation (AKTP^R270H^ and AKTP^Null^, respectively). Using these organoids, we found that the activation level of Wnt/β-catenin signaling is significantly higher in AKTP^R270H^ cells compared with AKTP^Null^ cells. Notably, Wnt activation in the AKTP^Null^ cells was significantly increased when co-cultured with AKTP^R270H^ cells. Expression analysis revealed that COX-2 is significantly upregulated in AKTP^R270H^ but not in AKTP^Null^ cells, suggesting that mutant p53 induces the COX-2/prostaglandin E2 (PGE2) pathway. Importantly, Wnt activation in co-cultured AKTP^Null^ cells with AKTP^R270H^ was significantly suppressed when treated with the inhibitor of COX-2 or PGE2 receptors EP2/EP4. Furthermore, stimulation with PGE2 increased Wnt signaling activity in AKTP^Null^ cells. These results indicate that the COX-2/PGE2 pathway is activated in the p53-stabilized cells in the missense-type p53-mutant cancer, and secreted PGE2 may transactivate Wnt/β-catenin signaling in neighboring p53-destabilized tumor cells in the intratumor microenvironment. Therefore, targeting stabilized mutant p53 or the COX-2/PGE2 pathway may suppress Wnt/β-catenin signaling of both mutant p53–stabilized and –destabilized cells; thus, this can be a possible preventive or therapeutic strategy.

**Significance:**

There is intratumor heterogeneity in the stabilization of missense mutant p53, and it has been thought that only cells with nuclear accumulation of mutant p53 have oncogenic function. However, using mouse intestinal tumor–derived organoids, we show that mutant p53–stabilized cells transactivate Wnt/β-catenin signaling in neighboring p53-destabilized cells through activating the COX-2/PGE2 pathway. These results suggest that both p53-stabilized cells and p53-destabilized cells contribute to malignant progression through interaction within the intratumor microenvironment.

## Introduction

*TP53* is one of the most frequently mutated genes in a wide range of cancers, including colorectal cancer (1, 2), and the *TP53* mutations were found in about 80% of colorectal cancer cases that are associated with metastasis (3). These results suggest that *TP53* mutation plays an important role in the late stage of cancer progression. The target molecules of p53 regulate a variety of biological processes, including cell-cycle arrest, DNA repair, and senescence, which may prevent tumor progression as a tumor suppressor (4). On the other hand, approximately 75% of *TP53* mutations in cancer are missense mutations in hot spots, resulting in the expression of mutant p53 with single amino acid substitutions, and such mutant p53 is thought to acquire a novel oncogenic function (5, 6).

Such gain of function of mutant p53 has been demonstrated in mouse genetic studies (7, 8). *Trp53*-null mutant mice spontaneously develop soft-tissue sarcomas and lymphomas, whereas mice expressing missense-type mutant p53 develop adenocarcinomas in the intestine and lung. It has also been reported that mutant p53 induces stem cell properties in breast and colorectal cancer cells (9, 10). In addition, we showed that the *Trp53*^R270H^ missense mutation (corresponding to human *TP53*^R273H^) causes submucosal invasion of intestinal tumors in *Apc*^Δ716^-mutant mice through activation of the inflammatory and Wnt signaling pathways (11). We also constructed a malignant intestinal tumor model by introducing *Apc*^Δ716^, *Kras*^G12D^, *Tgfbr2*^−/−^, and *Trp53*^R270H^ mutations to generate quadruple-mutant mouse model (12) and showed that missense-type mutant p53 promotes the survival and clonal expansion ability of single cell–dissociated cancer cells, which may contribute to colonization of disseminated cells in distant organs (13). It has also been reported that *in vivo* growth of *Trp53*^R248Q^ mouse tumors depends on sustained expression of mutant p53 (14). Similar results also showed that ablation of mutant *p53*^R248Q^ suppressed growth and invasion of mutant p53–driven tumors (15). These results suggest a possibility of gain-of-function mutant p53 as a therapeutic target molecule.

Although the genetic evidence for gain of function of mutant p53 has been accumulated, the underlying mechanisms of mutant p53–induced tumor promotion have not been fully understood yet. It has been shown that missense mutant p53 dramatically alters the transcriptome in tumor cells through modification of chromatin regulation by upregulation of chromatin regulatory genes or interaction with the SWI/SNF complex (16, 17). However, the precise molecular mechanisms of how mutant p53 promotes malignant phenotypes are still unclear. Moreover, there is intratumor heterogeneity in stabilization of mutant p53 (18), and it has not yet been understood whether there is interaction in oncogenic function between p53-stabilized and -destabilized cells.

In this study, we used mouse intestinal tumor–derived organoids that carried *Apc*, *Kras*, and *Tgfbr2* mutations together with *Trp53*^R270H^ or *Trp53*^−/−^ mutation to identify a tumor-promoting mechanism of mutant p53 and interaction between mutant p53-stabilized and -destabilized cells. Through the organoid experiments, we found that mutant *p53*^R270H^–expressing cells transactivate Wnt/β-catenin signaling of co-cultured *Trp53*^−/−^ cancer cells through induction of the COX-2/prostaglandin E2 (PGE2) pathway. Accordingly, these results suggest that the COX-2/PGE2 pathway induced by stabilized mutant p53 activates Wnt/β-catenin signaling in neighboring p53-destabilized cells, which may contribute to malignant progression of tumor tissues.

## Materials and Methods

### Organoid lines and cell culture experiments

An AKTP^R270H^ organoid was established from intestinal tumors of quadruple mutant *Apc*^Δ716^ (A), *Kras*^G12D^ (K) *Tgfbr2*^−/−^ (T), and *Trp53*^R270H^ (P) mice (12). AKTP^Null^ organoids were generated by the disruption of wild-type (WT) *Trp53* in AKT triple-mutant cells using the p53 CRISPR/Cas9 plasmid (13). *Trp53*-disrupted cells were selected with 10 µmol/L nutlin-3 (Cayman Chemical). These organoid cells were cultured on two-dimensional dishes with Advanced DMEM/F-12 medium (Gibco) supplemented with 10% FBS, 5 µmol/L anaplastic lymphoma kinase inhibitor (A83-01; Tocris Bioscience), 5 µmol/L GSK inhibitor (CHIR99021; Tocris Bioscience), and 10 µmol/L ROCK inhibitor (Y27632; Wako).

For suppression experiment of *Trp53*^R270H^ in AKTP^R270H^ cells, siRNA for *Trp53* or control-siRNA (Silencer select predesigned siRNA, Ambion, Thermo Fisher Scientific) and siRNA for *Trp53* (ON-TARGETplus Mouse *Trp53* siRNA-SMART pool, Dharmacon, Horizon) were transfected by Lipofectamine RNAiMAX (Thermo Fisher Scientific). Knockdown efficiency was examined by immunoblotting. Wnt signaling activation was examined after 9 hours of siRNA transfection.

For co-culture experiments, 2 × 10^2^ AKTP^Null^ and AKTP^R270H^ cells were mixed and co-cultured, or 4 × 10^2^ AKTP^Null^ or AKTP^R270H^ cells were cultured (monoculture) on Ultra-Low Attachment culture plates (Corning). After 72 hours, the size of organoids was measured using ImageJ (NIH) on the photographs. All cell culture experiments were repeated three times unless otherwise noted.

### Cell lines

HCT116 (#CCL-247, RRID: CVCL_0291), SW480 (#CCL-228, RRID: CVCL_0546), SW620 (#CCL-227, RRID: CVCL_0547), and Caco-2 (#HTB-37, RRID: CVCL_0025) were obtained from ATCC. LOVO (RCB1639, RRID: CVCL_0399) and COLO320 (RCB1193, RRID: CVCL_1989) were obtained from RIKEN BRC. Cells were maintained in high-glucose DMEM (Gibco) supplemented with 100 U/mL penicillin, 100 μg/mL streptomycin (Lonza), and 10% FBS (HyClone). Cells were maintained in the culture for maximum of 15 passages. Cells were monitored monthly for *Mycoplasma* contamination, and cell lines were authenticated using short tandem repeat analysis (Labcorp).

### Reporter assays

For measurement of Wnt signaling activity, the cells were transfected with Super 8× TOPFlash (RRID: Addgene_12456) or Super 8× FOPFlash (RRID: Addgene_12457; Addgene) using Lipofectamine LTX (Thermo Fisher Scientific). After 24 hours of transfection, the luciferase activity was measured using a Dual-Luciferase Reporter Assay system (Promega). For measurement of NF-κB activation, the cells were transfected with a pNL3.2.NF-κB-RE vector (Promega) using Lipofectamine LTX (Thermo Fisher Scientific). The luciferase activity was measured using a Nano-Glo Dual-Luciferase Reporter Assay System (Promega).

For measurement of Wnt signaling activity in co-culture experiment, AKTP^Null^ cells were transfected with Super 8× TOPFlash (RRID: Addgene_12456) or Super 8× FOPFlash (RRID: Addgene_12457). At 24 hours after transfection, the culture medium was changed, and then AKTP^Null^ cells were co-cultured with AKTP^R270H^ cells. After 24 hours of co-culture, the luciferase activity was measured.

### Inhibitors

For COX-2 inhibition, cells were cultured with 5 µmol/L celecoxib (Selleck Chemicals). For EP2 and EP4 inhibition, cells were cultured with 100 nmol/L PF-04418948 (Selleck Chemicals) or 10 µmol/L RQ-15986/CJ-042794 (AskAt Inc.), respectively (19). For PGE2 stimulation, 1 µmol/L PGE2 (Cayman Chemical) was added at 9 hours after transfection of the reporter vector.

### TOP-Venus reporter and EdU labeling experiments

Venus cDNA was replaced with luciferase cDNA of the TOPFlash vector (Upstate) to generate TOP-Venus and transfected to AKTP^Null^ cells using Lipofectamine LTX (Thermo Fisher Scientific). Transfectant TOP-Venus AKTP^Null^ cells were selected with 200 to 500 ng/mL puromycin. A total of 2 × 10^2^ TOP-Venus AKTP^Null^ and AKTP^R270H^ cells were mixed and co-cultured, or 4 × 10^2^ TOP-Venus AKTP^Null^ cells were monocultured on Ultra-Low Attachment culture plates (Corning). After 72 hours of culture, spheroids were picked up and embedded in Matrigel (Corning), and after another 72 hours of culture, spheroids were immunostained.

For 5-ethynyl-2'-deoxyuridine (EdU) labeling, we used 2 × 10^2^ AKTP^Null^ cells and Venus-labeled AKTP^R270H^ cells that were previously described (20). These cells were co-cultured on Ultra-Low Attachment culture plates (Corning). After 48 hours of culture, cells were treated with 10 mmol/L EdU for 90 minutes. After fixation with 4% paraformaldehyde, organoids were embedded in Matrigel, and EdU-labeled AKTP^Null^ cells were detected using Click-iT EdU cell proliferation assays (Thermo Fisher Scientific) and a confocal microscope (TCS SP8, Leica Microsystems). The numbers of EdU-labeled cells were counted at three different horizontal levels of each organoid, and the mean EdU labeling index was calculated.

### *Ctnnb1* short hairpin RNA experiments

Short hairpin RNA (shRNA) lentiviral vectors for *Ctnnb1* in pLKO.1-puro (MISSION shRNA, Sigma-Aldrich) were co-transfected to HEK293T cells with pCMV-VSV-G (Addgene, #8485, RRID: Addgene_8454) and pCMV-dR8.2 dvpr (Addgene, #8455, RRID: Addgene_8455) using PEI MAX (Polysciences) for packaging. Lentiviruses were infected to AKTP cells using polybrene (Sigma-Aldrich), and shRNA-expressing cells were selected with puromycin. Cell growth of shRNA lentivirus–infected cells was examined using CellTiter-Glo 2.0 Cell Viability Assay (Promega).

### Animal experiments

C57BL/6 mice (male; 6 weeks of age) were purchased (The Jackson Laboratory). AKTP^R270H^ or AKTP^Null^ organoids were treated with trypsin, and 3 to 5 × 10^5^ cells were injected into the mouse spleen. Liver metastatic tumors were examined at 4 weeks after the injection (*n* = 3) as follows. All animal experiments were carried out according to the protocol approved by the Committee on Animal Experimentation of Kanazawa University, Japan.

### Histology and IHC

Whole-liver tissues were collected from C57BL/6 mice. Human colorectal cancer samples were obtained from patients who underwent surgical resection at Ishikawa Prefectural Central Hospital, Japan. The experiments using human tissues were approved by the Human Genome/Gene Analysis Research Ethics Committee of Kanazawa University at #2021-002-603, and written informed consent was obtained from the patients. The tissues were fixated in 4% paraformaldehyde, paraffin-embedded, and sectioned at 4 μm thickness. For IHC, antibodies against E-cadherin (R&D Systems, Cat. #AF748, RRID: AB_355568), mouse p53 (Leica Biosystems, CM5, Cat. #NCL-p53-CM5p, RRID: AB_563933; Cell Signaling Technology, 1C12, Cat. #2524, RRID: AB_331743), human p53 (Santa Cruz Biotechnology, DO7, Cat. #47698, RRID: AB_628083), β-catenin (Cell Signaling Technology, D10A8, #8480, RRID: AB_11127855), and COX-2 (Cayman Chemicals, polyclonal antibody, aa584-598, RRID: AB_10079419) were used as primary antibodies. Staining signals were visualized using the VECTASTAIN Elite Kit (Vector Laboratories). For fluorescence IHC, Alexa Fluor 594– or Alexa Fluor 488–conjugated antibodies (Molecular Probes, Fluor 594, #A21207, RRID: AB_141637; Fluor 488, #A21206, RRID: AB_2535792) were used as the second antibody. The immunostained specimens were examined using a confocal microscope (Leica TCS SP8, Leica Microsystems). The number of p53-stabilized and β-catenin nuclear-accumulated cells were counted in three to four independent microscopic fields, and the mean number per tumor was calculated.

### Organoid immunocytochemistry

The organoids were immunostained using antibodies against mouse p53 (Leica Biosystems, CM5, Cat. #NCL-p53-CM5p, RRID: AB_563933; Cell Signaling Technology, 1C12, Cat. #2524, RRID: AB_331743) and COX-2 (Cayman Chemicals, polyclonal antibody, aa584-598, RRID: AB_10079419) and Alexa Fluor 594– or Alexa Fluor 488–conjugated antibodies (Molecular Probes, Fluor 594, RRID: AB_141637; Fluor 488, RRID: AB_2535792) for the secondary antibody. The immunostained organoids were examined using a confocal microscope (Leica TCS SP8, Leica Microsystems).

### Immunoblotting

Organoid cells were lysed in lysis buffer, and protein samples were separated in 10% SDS-PAGE. Antibodies against mouse p53 (Cell Signaling Technology, 1C12, Cat. #2524, RRID: AB_331743) and COX-2 (Cayman Chemicals, polyclonal antibody, aa584-598, RRID: AB_10079419) were used. Antibodies for GAPDH (FUJIFILM Wako, Cat. #016-25523, RRID: AB_2814991) and β-actin (Wako, 6D1, #010-27841, RRID: AB_2858279) were used as the internal control. The enhanced chemiluminescence detection system (GE Healthcare) was used to detect the signals.

### *Trp53* copy-number analysis

*Trp53* genotyping in AKTP^R270H^ cell lines was performed using a TaqMan SNP Genotyping assay (Thermo Fisher Scientific). The custom TaqMan probes for WT *Trp53* (VIC dye) and mutant *Trp53*^R270H^ (FAM dye) were designed in exon 8 including the R270 codon of mouse *Trp53*. Sequences of TaqMan probes are as follows:

Forward primer: CCG​GAT​AGT​GGG​AAC​CTT​CTG

Reverse primer: TCT​TCT​GTA​CGG​CGG​TCT​CT

The TaqMan probe to detect WT *Trp53* (VIC dye) is CTT​TGA​GGT​TC**G**TGT​TTG​T, and the TaqMan probe to detect mutant *Trp53*^R270H^ (FAM dye) is TTT​GAG​GTT​C**A**TGT​TTG​T. The PCR results were analyzed using Allele discrimination in Agilent AriaMx (Agilent Technologies).

### Public database for RNA sequencing

For RNA sequencing (RNA-seq) data of AKTP^R270H^ and AKTP^Null^ organoids and *AKTFP*^R270H/LOH^ and *AKTFP*^R270H/+^ organoids, we used public datasets of the DNA Data Bank of Japan (accessions #PRJDB5631 and #PRJDB15570).

### Statistical analysis

**Figure 1:**
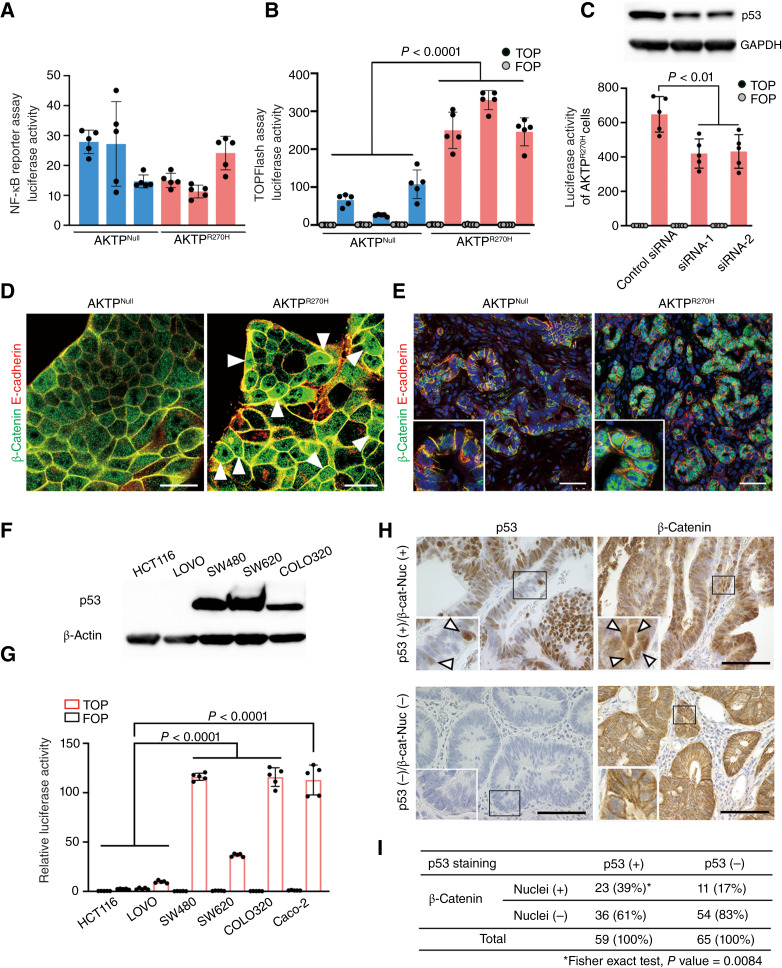
Promotion of Wnt/β-catenin signaling in missense-type p53-mutant colon tumors. **A,** Luciferase activities of NF-κΒ reporter assay are shown as a bar graph (mean ± SD). **B,** Luciferase activities of Wnt/β-catenin reporter assay (TOPFlash) are shown as a bar graph (mean ± SD). Three independent AKTP^Null^ and AKTP^R270H^ organoid lines for each were used in the reporter assays (**A** and **B**). Statistical analysis was performed using the one-way ANOVA test (**A** and **B**). **C,** Luciferase activities of Wnt/β-catenin reporter assay for control-transfected and *Trp53* siRNA–transfected AKTP^R270H^ organoid cells are shown as a bar graph (mean ± SD). The immunoblotting result for p53 is shown. GAPDH was used as the internal control. Each dot in **A**–**C** graphs indicates an independent experiment. *P* values are indicated. **D,** Representative confocal microscopy images of fluorescence immunocytochemistry for β-catenin (green) and E-cadherin (red) in AKTP^Null^ and AKTP^R270H^ organoids. Arrowheads indicate cells with β-catenin stabilization. Bars, 25 μm. **E,** Representative photographs of fluorescence IHC for β-catenin (green) and E-cadherin (red) of AKTP^Null^ and AKTP^R270H^ liver metastatic tumors. Insets show enlarged images. Bars, 100 μm. Note that β-catenin accumulation was predominantly found in AKTP^R270H^ tumors. **F,** Immunoblotting results for p53 in colorectal cancer cell lines. β-Actin was used as the internal control. **G,** Luciferase activities of Wnt/β-catenin reporter assay (TOPFlash) for colorectal cancer cell lines are shown as a bar graph (mean ± SD). Each dot indicates an independent experiment. The *P* value is provided. **H,** Representative photographs of immunostaining for p53 (left) and β-catenin (right) of p53-positive and β-catenin nuclear-accumulated (top) and p53-negative and β-catenin not nuclear–accumulated (bottom) human colorectal cancer. Insets show enlarged images of the boxed area. Arrowheads indicate p53 stabilization (left) and β-catenin nuclear accumulation (right). Bars, 200 μm. **I,** Ratio of β-catenin nuclear accumulation in p53-positive (+) and p53-negative (−) colorectal cancer scored using the IHC results in **H**. The *P* value is indicated.

The data were analyzed using two-sided unpaired *t* tests unless otherwise mentioned and presented as the means ± SD. Statistical analyses for Fig. 1A, B, and G were performed using a one-way ANOVA test. The Fisher exact test was used for Fig. 1G. A value of *P* < 0.05 was considered statistically significant. GraphPad Prism 9 (GraphPad) was used for statistical analyses. All data were reproduced with at least two independent experiments and at least three biological replicates.

### Data availability

The data generated in this study are available upon request from the corresponding authors.

## Results

### Missense mutant p53 promotes Wnt/β-catenin signaling in intestinal tumors

We previously established an intestinal tumor–derived organoid, AKTP, in which *Apc*^Δ716^, *Kras*^G12D^, *Tgfbr2*^−/−^, and *Trp53*^R270H^ mutations were introduced (12). To investigate the role of oncogenic function of mutant *p53*^R270H^, we further constructed *Trp53*^−/−^ AKTP organoid by disrupting *Trp53* using the CRISPR/Cas9 (13). Here, we named AKTP organoid lines with *Trp53*^R270H^ or *Trp53*^−/−^ mutations as AKTP^R270H^ or AKTP^Null^, respectively. We used three independently established AKTP^R270H^ lines in this study, and genomic PCR analysis revealed that all lines had two copies of the *Trp53*^R270H^ mutant alleles, indicating the loss of WT *Trp53* by copy-number neutral loss of heterozygosity (LOH; Supplementary Fig. S1).

We have previously shown that the inflammatory pathway and Wnt/β-catenin signaling are activated in the *Apc*^Δ716^*Trp53*^R270H^ double-mutant intestinal tumor–derived organoids compared with that in the simple *Apc*^Δ716^-mutant tumor organoids (11). Therefore, we examined the activation levels of NF-κB in the inflammatory pathway and Wnt/β-catenin signaling in the AKTP cells using reporter assays. We found that NF-κB activation was at the similar levels between AKTP^R270H^ and AKTP^Null^ organoid cells (Fig. 1A). In contrast, the levels of Wnt/β-catenin signaling were significantly higher in all AKTP^R270H^ compared with AKTP^Null^ organoid lines (Fig. 1B). Moreover, transfection of siRNA against *Trp53* mRNA into AKTP^R270H^ cells significantly suppressed Wnt signaling activation with a decrease in p53 expression levels (Fig. 1C). These results indicate that mutant *p53*^R270H^ promotes Wnt/β-catenin signaling activation.

We confirmed *p53*^R270H^-induced Wnt signaling activation by other methods. First, we performed an Ingenuity Pathway Analysis using RNA-seq data of AKTP^R270H^ and AKTP^Null^ organoids (13) and found that the Wnt signaling pathways (CTNNB1 and TCF7L2 pathways) were significantly activated in AKTP^R270H^ (Supplementary Fig. S2). In addition, immunostaining of cultured AKTP^R270H^ organoids and histologic sections of liver metastasis of AKTP^R270H^ organoid cells showed β-catenin stabilization and nuclear accumulation, which were rarely found in AKTP^Null^ organoid cultures and metastatic tissues (Fig. 1D and E).

We further examined the relationships between mutant p53 and Wnt/β-catenin signaling using *TP53* WT and *TP53*-mutant human colorectal cancer cell lines (21). As expected, stabilized p53 was detected in the missense-type *TP53*-mutant SW480, SW620, and COLO320 cells but not in *TP53* WT HCT116 and LOVO cells (Fig. 1F). Notably, Wnt/β-catenin signaling is significantly higher in *TP53*-mutant cells than in *TP53* WT cells (Fig. 1G). However, the Wnt/β-catenin signaling is also high in the *TP53*-null mutant Caco-2 cells (22). We further examined the primary colorectal cancer tissues by IHC using tissue microarray. In the p53 staining–positive colorectal cancer group, β-catenin nuclear accumulation was found in 39% of tissues, whereas the ratio was significantly decreased to 17% in p53-negative colorectal cancer tissues (Fig. 1H and I). These results collectively suggest that the Wnt signaling activation in human colorectal cancer may involve, though not exclusively, the presence of missense-type mutant p53, similar to that found in mouse intestinal tumors.

### Missense mutant p53 promotes Wnt/β-catenin signaling in a non–cell autonomous mechanism

**Figure 2:**
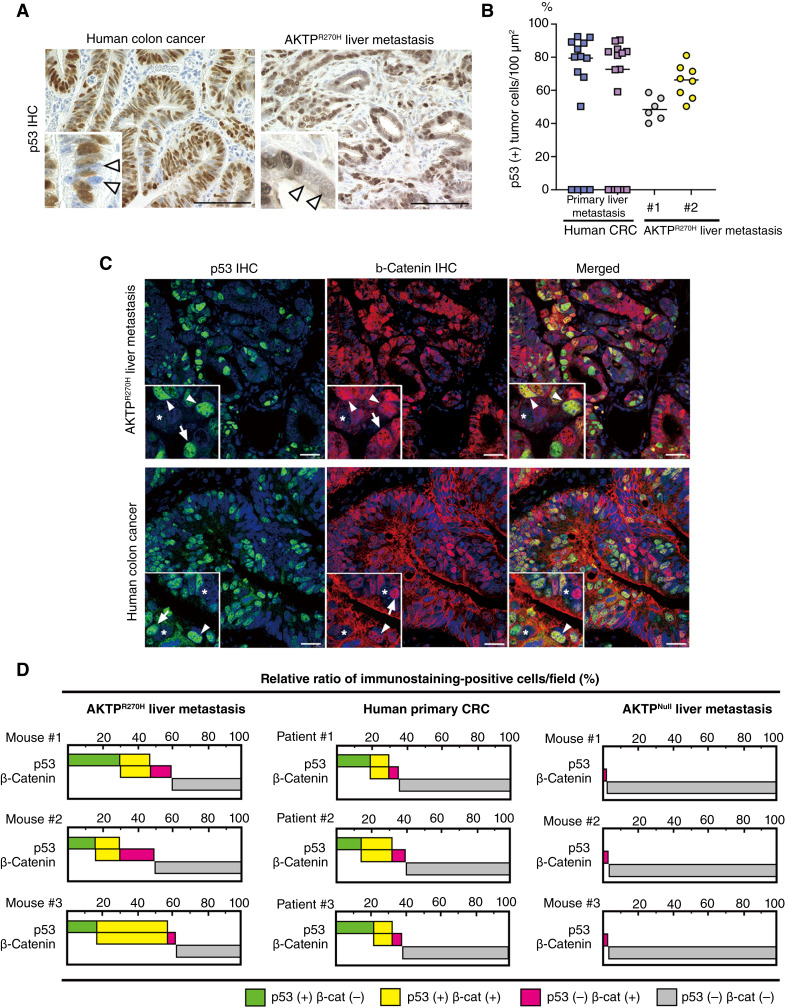
Heterogeneity of p53 and β-catenin stabilization in tumor cells. **A,** Representative photographs of IHC for p53 of human colon cancer (left) and AKTP^R270H^ liver metastatic tumor (right). Insets show enlarged images. Arrowheads indicate p53-negative (destabilized) tumor cells. Bars, 100 μm. **B,** Ratio of p53-positive (+) cells in primary and metastatic colorectal cancer (CRC; squares) and AKTP^R270H^ liver metastatic tumors of two independent lines (circles). Each dot indicates the results of the independent microscopic fields. **C,** Representative photographs of fluorescence IHC for p53 (green, left), β-catenin (red, center), and merged images (right) of AKTP^R270H^ liver metastasis (top) and human colon cancer (bottom) Insets show enlarged images. Arrowheads and arrows indicate double-positive and single-positive cells, respectively. Asterisks indicate double-negative cells. Bars, 100 μm. **D,** Relative proportions (%) of p53-positive, β-catenin–positive, and double-negative cells in the liver metastasis of AKTP^R270H^ cells (left), AKTP^Null^ cells (right), and human colorectal cancer (center) are shown as bar graphs. Three biologically independent data for each are shown.

It has been reported that there is heterogeneity in p53 stabilization among *TP53*-mutant cells (18). We also found the heterogeneity in p53 stabilization in both human colorectal cancer and mouse AKTP^R270H^ liver metastasis by IHC (Fig. 2A). In p53-positive human colorectal cancer, p53 stabilization was found in approximately 80% and 72% of tumor cells in the primary and liver metastases, respectively (Fig. 2A and B). Similarly, in AKTP^R270H^ metastatic tumors, p53 stabilization was found in approximately 50% to 66% of two different cell lines in average. Specifically, not all cancer cells with p53 mutations have stabilized p53 protein, and there is intratumor heterogeneity in p53 stabilization. As stabilization of mutant p53 is thought to be a prerequisite for the oncogenic function, these results suggest that there is intratumor heterogeneity in the oncogenic function of mutant p53 in the p53-mutant tumors.

We therefore examined whether the stabilization of p53 and β-catenin occurs simultaneously in the same cells or not by double-fluorescence immunostaining. In the AKTP^R270H^ metastatic tumors, we found double-positive cells for β-catenin and p53 as expected; however, we also found certain populations of single-positive cells for β-catenin or p53 [Fig. 2C (top) and D (left)]. Similar immunostaining patterns, namely, double-positive and single-positive cells for p53 and β-catenin were also found in human colorectal cancer tissues [Fig. 2C (bottom) and D (center)]. In contrast, nuclear accumulation of β-catenin was rarely detected in AKTP^Null^ metastatic tumors [Fig. 2D (right)]. Taken together, these results suggest that mutant p53 activates Wnt/β-catenin signaling not only in the p53-stabilized cells but also in the p53-destabilized cells possibly through a non–cell autonomous mechanism.

### Mutant p53–induced COX-2/PGE2 pathway transactivates Wnt/β-catenin signaling in p53-null/destabilized cells

**Figure 3:**
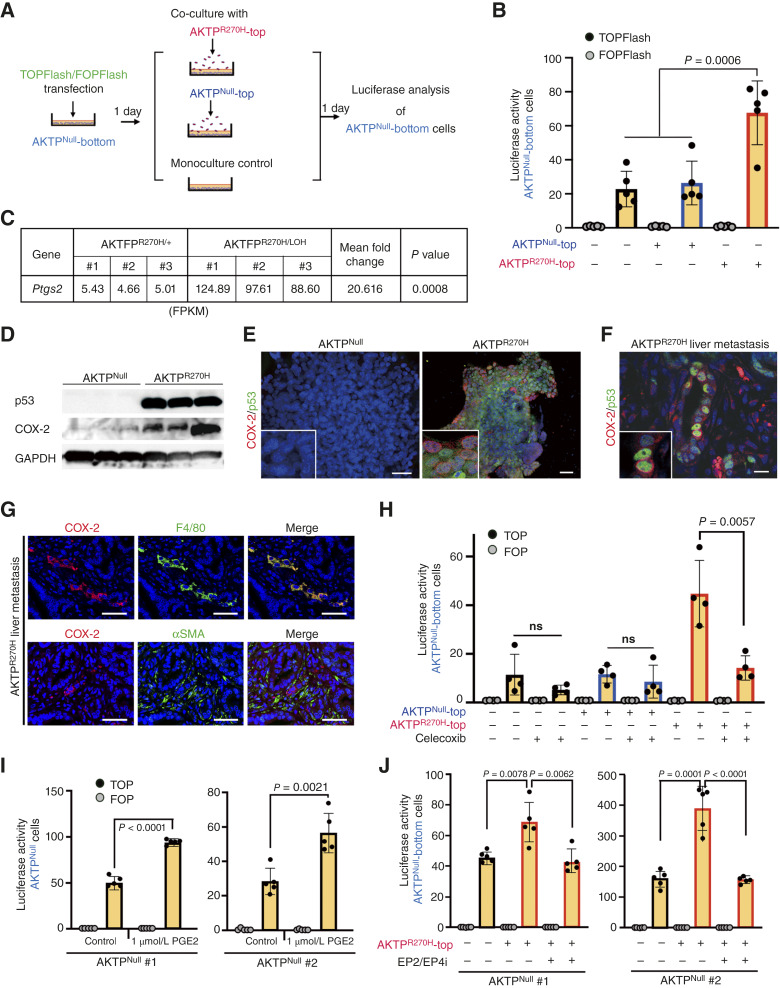
Activation of Wnt/β-catenin signaling in p53-negative cells through the COX-2/PGE2 pathway. **A,** Schematic drawing of Wnt/β-catenin reporter assays (TOPFlash) of co-cultured AKTP^Null^-bottom cells with AKTP^R270H^-top cells or AKTP^Null^-top cells. **B,** Luciferase activities of Wnt reporter assays (TOPFlash) of AKTP^Null^-bottom cells co-cultured with AKTP^Null^-top cells or AKTP^R270H^-top cells are shown as a bar graph (mean ± SD). Each dot represents an independent experiment. The *P* value is provided. **C,** RNA-seq data for *Ptgs2* in three independent AKTFP^R270H/+^ and AKTFP^R270H/LOH^ organoid lines. fragments per kilobase of exon per million reads mapped, the mean fold change, and *P* value are provided. **D,** Immunoblotting results for p53 and COX-2 in three independent AKTP^Null^ and AKTP^R270H^ lines. GAPDH was used as the internal control. **E,** Representative fluorescence immunocytochemistry images for p53 (green) and COX-2 (red) of AKTP^Null^ (left) and AKTP^R270H^ (right) organoids. **F,** Representative image of fluorescence IHC for p53 (green) and COX-2 (red) of AKTP^R270H^ liver metastatic tumor. Insets in **E** and **F** show enlarged images. Bars in **E** and **F**, 100 μm. **G,** Representative images of fluorescence IHC for COX-2 (red) and F4/80 (macrophages, green) or αSMA (myofibroblasts, green). Merged images are shown (right). Bars, 50 μm. **H,** Luciferase activities of Wnt reporter assays (TOPFlash) of AKTP^Null^-bottom cells in co-cultured AKTP^Null^-top or AKTP^R270H^-top cells or control AKTP^Null^-bottom monoculture cells in the presence or absence of COX-2 inhibitor, celecoxib, are shown as a bar graph (mean ± SD). Each dot represents an independent experiment. The *P* value is indicated. ns, not significant. **I** and **J,** Luciferase activities of Wnt/β-catenin reporter assays in AKTP^Null^ cells treated with PGE2 (**I**) or AKTP^Null^ cells co-cultured with AKTP^R270H^ treated with EP2/EP4 inhibitors (**J**) are shown as bar graphs (mean ± SD). Data from two independent AKTP^Null^ lines (#1 and #2) are shown. Each dot indicates an independent experiment. *P* values are provided.

To investigate a possible mechanism for Wnt signaling activation in cancer cells in which the mutant p53 protein is destabilized and undetectable, we used AKTP^Null^ as a model for these p53-destabilized cells in the p53-mutant tumors. After co-culture with AKTP^R270H^ cells, we examined the Wnt signaling activity in AKTP^Null^ cells using TOPFlash reporter assay (Fig. 3A). AKTP^Null^ cells (AKTP^Null^-bottom cells in Fig. 3B) carry an *Apc* mutation; thus, reporter assay showed basal activation of Wnt signaling (Fig. 3B). Importantly, the TOPFlash level in AKTP^Null^-bottom cells was significantly increased when co-cultured with AKTP^R270H^-top cells compared with that in the co-culture with AKTP^Null^-top cells or the monoculture control, suggesting a paracrine mechanism for Wnt activation in AKTP^Null^ cells by AKTP^R270H^ cells (Fig. 3B).

We previously performed RNA-seq of AKTFP^R270H/LOH^ (*Trp53*^R270H^ with loss of WT *Trp53* by LOH) and AKTFP^R270H/+^ (*Trp53*^*R270H/+*^ heterozygous) organoids and found that the expression level of *Ptgs2* encoding COX-2 was significantly upregulated in AKTFP^R270H/LOH^ cells (Fig. 3C; ref. 23). As the mutant p53 stabilization is significantly increased by loss of WT p53 by LOH (24, 25), the expression data suggest that COX-2 upregulation is caused by stabilized mutant p53 function. As expected, we confirmed increased levels of COX-2 expression in all AKTP^R270H^ organoid lines by immunoblotting compared with those in AKTP^Null^ cells (Fig. 3D). Furthermore, we confirmed COX-2 expression in AKTP^R270H^ cells by immunostaining, although the levels of COX-2 expression seemed to vary (Fig. 3E). On the other hand, COX-2 was not detected in AKTP^Null^ cells. Liver metastatic tumors of AKTP^R270H^ cells also showed COX-2 expression in p53-stabilized tumor cells (Fig. 3F). We also found COX-2–expressing macrophages but not α-smooth muscle actin (α-SMA)-expressing myofibroblasts in the tumor stroma of the liver metastatic tumors of AKTP^R270H^ cells (Fig. 3G). COX-2 is a rate-limiting enzyme for prostaglandin biosynthesis, and several studies have shown an interaction between the COX-2/PGE2 pathway and Wnt/β-catenin signaling activation (26–28). Importantly, treatment with celecoxib, a selective COX-2 inhibitor, dramatically suppressed Wnt/β-catenin signaling activation in AKTP^Null^-bottom cells co-cultured with AKTP^R270H^-top cells (Fig. 3H). In contrast, direct stimulation of AKTP^Null^ cells with PGE2 significantly increased Wnt signaling activity (Fig. 3I). Furthermore, suppression of PGE2 signaling by treating cells with the EP2/EP4 receptor inhibitor suppressed Wnt signaling activation in AKTP^Null^-bottom cells that were co-cultured with AKTP^R270H^-top cells (Fig. 3J). The similar results were obtained in the two independently established AKTP^Null^ organoid lines (Fig. 3I and J). Accordingly, these results indicate that AKTP^R270H^ cells transactivate Wnt/β-catenin signaling in AKTP^Null^ cells through induction of the COX-2/PGE2 pathway, and PGE2 signaling through the EP2 and/or EP4 receptor is vital for Wnt activation in AKTP^Null^ cells. These results suggest that in p53-mutant tumors, cancer cells with stabilized mutant p53 activate Wnt/β-catenin signaling in neighboring p53-destabilized cells through the COX-2/PGE2 pathway. It is also possible that macrophage-secreted PGE2 contributes to Wnt/β-catenin signaling activation in p53-destabilized cells in the liver metastatic tumors, although it remains to be further investigated.

Interestingly, Wnt signaling activity in AKTP^R270H^ cells was not affected by treatment with the COX-2 inhibitor or EP2/EP4 inhibitor (Supplementary Fig. S3). We previously showed that missense p53 mutations resulted in Wnt/β-catenin pathway activation with increased promoter accessibility (11). It is possible that Wnt/β-catenin signaling is activated in the p53-stabilized AKTP^R270H^ cells by the COX-2/PGE2 pathway–independent mechanism.

### Mutant p53 promotes proliferation of p53-null/destabilized cells

**Figure 4:**
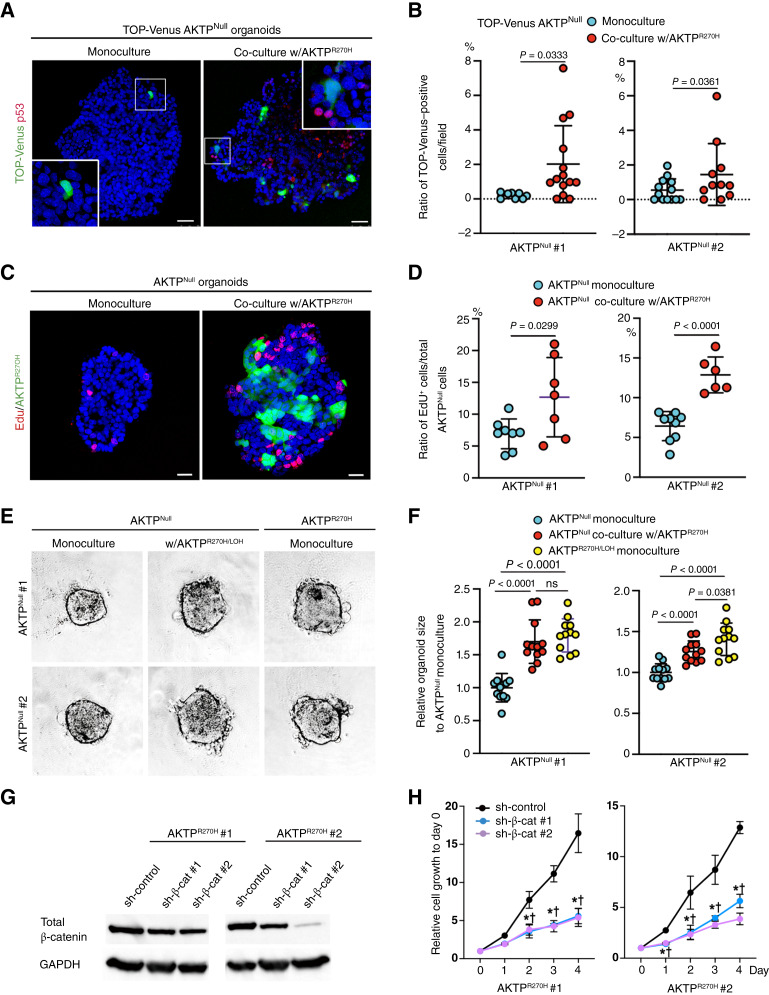
Promotion of cell proliferation of p53-negative tumor cells co-cultured with p53-mutant cells. **A,** Representative fluorescent immunocytochemistry images for p53 (red) and TOP-Venus–positive (Wnt/β-catenin–activated) cells (green) of monocultured AKTP^Null^ (left) and co-cultured AKTP^Null^ with AKTP^R270H^ (right) organoids. Insets show enlarged images. Bars, 50 μm. **B,** Ratios of TOP-Venus–positive (Wnt/β-catenin–activated) cells per microscopic field. Each dot indicates the result in the independent field. Data from two independent AKTP^Null^ lines (#1 and #2) are shown. *P* values are provided. **C,** Representative images of fluorescent immunocytochemistry for EdU-labeled cells (red) of monocultured AKTP^Null^ (left) and co-cultured AKTP^Null^ with Venus-labeled AKTP^R270H^ organoids (green; right). Bars, 25 μm. **D,** Ratios of EdU-labeled cells to total monocultured AKTP^Null^ cells or co-cultured AKTP^Null^ cells with AKTP^R270H^. Each dot indicates the results in the independent microscopic field. Data from two independent AKTP^Null^ lines (#1 and #2) are shown. *P* values are indicated. **E,** Representative photographs of monocultured AKTP^Null^ (left) and AKTP^R270H^ organoids (right) and co-cultured AKTP^Null^ and AKTP^R270H^ organoids (center). Results from two independent AKTP^Null^ lines (#1 and #2) are shown. Bars, 200 μm. **F,** Relative organoid sizes of monocultured AKTP^Null^ and AKTP^R270H^ cells and co-cultured AKTP^Null^ and AKTP^R270H^ cells. Each dot indicates the independent organoid size. Results of two independent AKTP^Null^ lines (#1 and #2) are shown. *P* values are shown. ns, not significant. **G,** Immunoblotting results for total β-catenin in two lines of sh-β-catenin–transfected AKTP^Null^ cells and sh-control–transfected cells. GAPDH was used as the internal control. **H,** Relative cell growth rate of sh-β-catenin–transfected AKTP^Null^ cells (#1 and #2) with sh-control–transfected cells. The results of two independent AKTP^R270H^ lines are shown (**G** and **H**). Asterisks and daggers, *P* < 0.05 vs. sh-control.

We further examined the Wnt signaling activity by imaging analysis using TOP-Venus reporter–transfected AKTP^Null^ cells that express Venus in response to Wnt/β-catenin signaling. Notably, the number of Venus-positive AKTP^Null^ cells increased significantly when co-cultured with AKTP^R270H^ cells compared with that of control AKTP^Null^ cell monocultures (Fig. 4A and B). Similar results were obtained in the two independent AKTP^Null^ organoid lines. These results confirmed that AKTP^R270H^ cells transactivate the Wnt signaling activity of AKTP^Null^ cells.

We next analyzed the cell proliferation rate of monocultured AKTP^Null^ cells and co-cultured AKTP^Null^ cells with AKTP^R270H^-Venus cells by EdU labeling assay. Fluorescent immunostaining of the organoids revealed that the number of EdU-labeled AKTP^Null^ cells increased significantly when co-cultured with AKTP^R270H^ cells compared with that of monocultured cells (Fig. 4C and D). The similar results were obtained in the two independent AKTP^Null^ organoid lines.

The size of organoids developed in the Matrigel was significantly larger in AKTP^R270H^ compared with AKTP^Null^ cells, indicating a higher proliferation rate of AKTP^R270H^ cells (Fig. 4E and F). Interestingly, the size of mixed organoids of AKTP^Null^ and AKTP^R270H^ cells was intermediate; however, it tended to be of similar size to AKTP^R270H^ cells at least in one AKTP^Null^ organoid line [Fig. 4F (left)]. These results indicate that AKTP^R270H^ cells promote the proliferation of AKTP^Null^ cells.

Finally, we examined a role of Wnt/β-catenin signaling in AKTP^R270H^ cell proliferation. Notably, partial suppression of β-catenin expression by shRNA transfection significantly suppressed proliferation in the independent AKTP^R270H^ organoid lines (Fig. 4G and H). Accordingly, it is possible that transactivation of Wnt/β-catenin signaling in AKTP^Null^ cells may contribute to increased proliferation.

## Discussion

Recently, it has been reported that removal of mutant *p53* genes in human cancer cells did not affect the proliferation and survival of cancer cells including colorectal cancer (29). On the other hand, we previously showed that mutant p53 enhances the clonal expansion and colony formation ability of the single cell–dissociated tumor cells, suggesting increased stemness of the p53-mutant cancer cells (13). As is generally thought, Wnt signaling is essential for the maintenance of tissue and tumor stem cells (30). Therefore, it is possible that mutant p53 plays an important role in colonization of the disseminated tumor cells in the liver through Wnt/β-catenin activation rather than growth of established tumors. Consistently, it has been reported that p53 mutations induce stem cell properties in cancer cells (9, 10).

Although the precise molecular mechanisms underlying the gain-of-function activity by mutant p53 remain unclear, previous studies have indicated the potential involvement of epigenetic modification by mutant p53, which resulted in significant transcriptome changes, including *VEGFR2* upregulation (16, 17). Additionally, our recent findings suggest that the upregulation of the transcription factor *Hmga2* by mutant p53 contributes to partial epithelial-to-mesenchymal transition and cluster migration of cancer cells (23). However, these mutant p53–induced oncogenic functions are considered cell intrinsic mechanisms. Namely, it was thought that only mutant p53–stabilized cells carry malignant phenotypes. However, in the present study, we demonstrate for the first time that mutant p53–stabilized cells may activate Wnt/β-catenin signaling of p53-destabilized cells by a paracrine mechanism. Therefore, it seems that mutant p53 enhances Wnt/β-catenin signaling of whole-tumor tissues through cell-autonomous and non–cell autonomous mechanisms.

**Figure 5:**
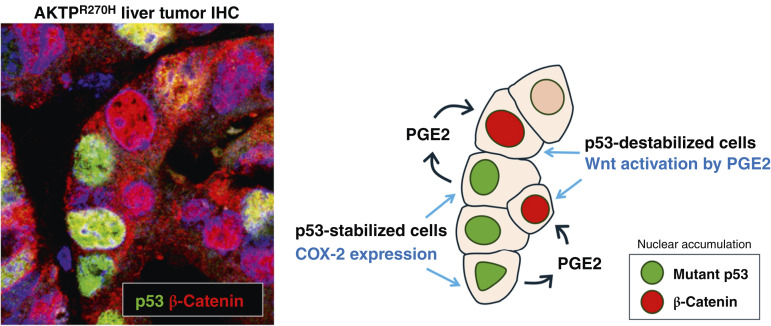
Schematic drawing of mutant p53–induced transactivation of Wnt/β-catenin signaling in p53-deficient cells. Double-fluorescent IHC for p53 and β-catenin of liver metastatic tumors of AKTP^R270H^ cells (left) and sketch of tumor cells from the photograph with the Wnt activation mechanism are shown (right).

In this study, we elucidated that mutant p53 induces COX-2 expression, and the downstream product PGE2 activates EP2/EP4 receptor signaling in surrounding p53-destabilized cells, leading to the activation of Wnt/β-catenin signaling (Fig. 5). Previously, we showed that the missense-type p53 mutation in the intestinal tumor cells leads to a dramatic transcriptomic shift through an increase in promoter accessibility (11), and inflammatory pathways, including TNFα/NF-κB, are significantly activated in p53-mutant AKTP^R270H^ cells (13). TNFα has been shown to induce COX-2 expression via NF-κB activation (31). Accordingly, it is possible that the TNFα/NF-κB pathway may be a potential driver of COX-2 induction in AKTP^R270H^ cells.

The COX-2/PGE2 pathway has been reported to activate Wnt signaling in colorectal cancer cells (26, 27), and the interaction between PGE2 and Wnt signaling has been shown to be important in the regulation of hematopoietic stem cells and organ regeneration processes (28). These results suggest that COX-2/PGE2-induced Wnt activation is thought to play an important role in the proliferation of colorectal cancers harboring p53 mutations. However, we could not elucidate the mechanism of PGE2-induced Wnt/β-catenin pathway activation in the present study. Both AKTP^R270H^ and AKTP^Null^ cells lack the WT *Apc* gene, resulting in constitutive stabilization of β-catenin. Accordingly, it is possible that PGE2 signaling may be involved in the regulation of nuclear localization or the degradation process of β-catenin, although it remains to be examined. In this study, we found that infiltrating macrophages in the metastatic tumor stroma also express COX-2. It is thus possible that macrophage-derived PGE2 may contribute to Wnt signaling activation in the p53-destabilized cancer cells, although this requires further investigation.

Accumulating evidence from genetic studies has revealed that the COX-2/PGE2 pathway plays a critical role in colorectal cancer development by generating an inflammatory microenvironment (32, 33). In addition, activation of the COX-2/PGE2 pathway in fibroblasts surrounding intestinal crypts has recently been reported to activate YAP via EP4 receptors on intestinal epithelial cells, thereby promoting tumorigenesis (34). These results suggest that activation of the COX-2/PGE2 pathway in mutant p53–stabilized cells may have significant effects on tumorigenesis possibly beyond activation of the Wnt/β-catenin pathway in the neighboring p53-destabilized cells. Accordingly, it is possible that the COX-2/PGE2 pathway is an effective therapeutic target against both p53-stabilized and -destabilized cells in *TP53*-mutant cancer.

## Supplementary Material

Supplementary Figure S1 Trp53 LOH analysis for AKTP R270H cells

Supplementary Figure S2 IPA results for AKTP R270 vs AKTP Null cells

Supplementary Figure S3 TOPFLash analysis in COX-2 or EP2/EP4 inhibitor-treated AKTP R270H cells
