# Supplementary material for: Missense Mutant p53 Transactivates Wnt/β-Catenin Signaling in Neighboring p53-Destabilized Cells through the COX-2/PGE2 Pathway
Source: Cancer Res Commun. 2025 Jan 3;5(1):13–23. doi: 10.1158/2767-9764.CRC-24-0471 (PMC11695814; doi:10.1158/2767-9764.CRC-24-0471)
Supplement: Supplementary Figure S1 — Trp53 LOH analysis for AKTP R270H cells [file crc-24-0471_supplementary_figure_s1_suppsf1.pdf]

Supplementary Figure S1

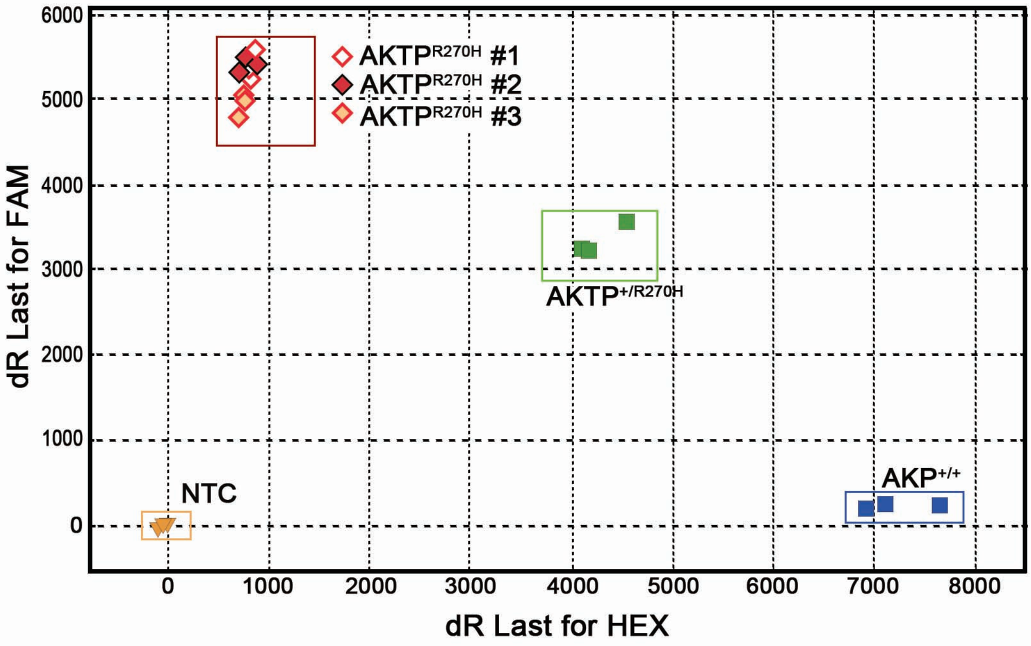

Supplementary Figure S1

Genotypes of *Trp53* in the AKTP^{R270H} cell lines. Genomic DNA from three independently established AKTP^{R270H} lines (#1~#3) was analyzed by TaqMan SNP genotyping. *Trp53*$^{+/R270H}$ heterozygous AKTP cells and *Trp53*$^{+/+}$ wild-type AKP cells were used as references. *Trp53* allelic discrimination data are shown as a scatter plot of *Trp53* wild-type codon 270 (CGT) (HEX dye) versus *Trp53* mutant codon R270H (CAT) (FAM dye). Note that all AKTP^{R270H} lines carry two copies of mutant *Trp53*^{R270H}. Template control (NTC) is shown as yellow triangles.
