# Supplementary material for: Missense Mutant p53 Transactivates Wnt/β-Catenin Signaling in Neighboring p53-Destabilized Cells through the COX-2/PGE2 Pathway
Source: Cancer Res Commun. 2025 Jan 3;5(1):13–23. doi: 10.1158/2767-9764.CRC-24-0471 (PMC11695814; doi:10.1158/2767-9764.CRC-24-0471)
Supplement: Supplementary Figure S2 — IPA results for AKTP R270 vs AKTP Null cells [file crc-24-0471_supplementary_figure_s2_suppsf2.pdf]

Supplementary Figure S2

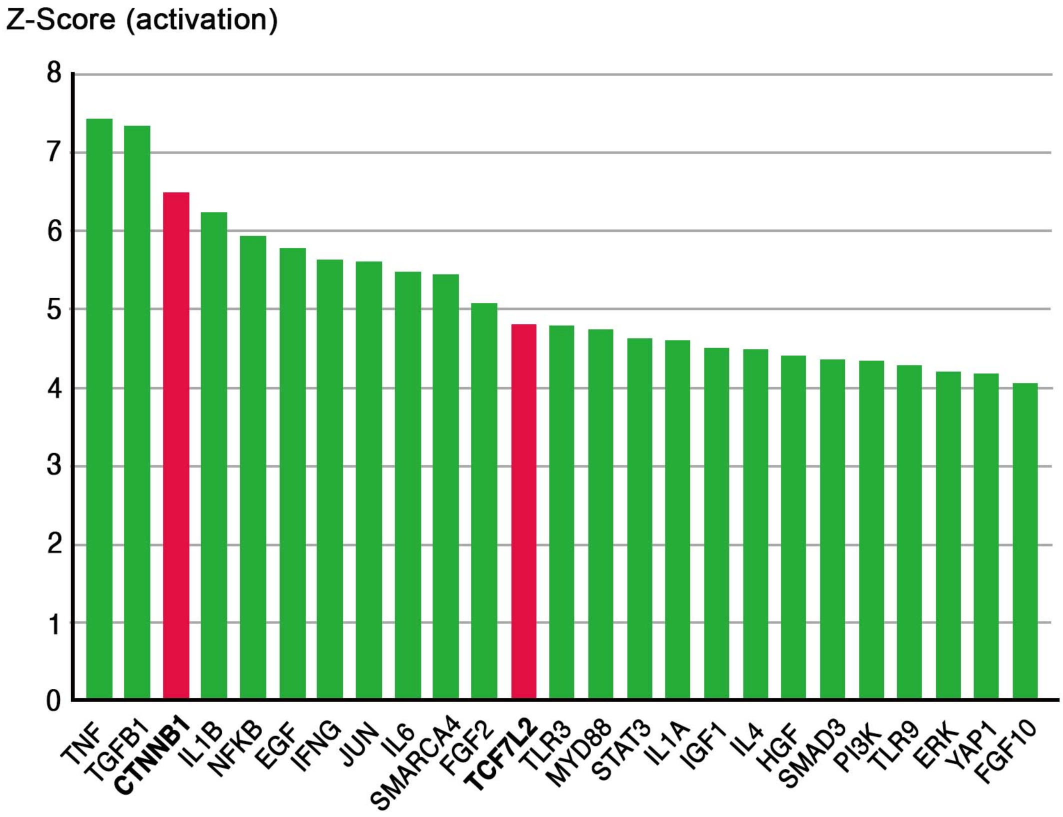

Supplementary Figure S2

Ingenuity Pathway Analysis (IPA) results using the upregulated gene set in $AKTP^{R270H}$ cells vs. $AKTP^{Null}$ cells are shown as a bar graph. Activated upstream pathways (z-score > 4) are listed. Note that Wnt pathways (CTNNB1 and TCF7L2, red bars) are significantly activated in $AKTP^{R270H}$ cells compared to $AKTP^{Null}$ cells.
