# Supplementary material for: Missense Mutant p53 Transactivates Wnt/β-Catenin Signaling in Neighboring p53-Destabilized Cells through the COX-2/PGE2 Pathway
Source: Cancer Res Commun. 2025 Jan 3;5(1):13–23. doi: 10.1158/2767-9764.CRC-24-0471 (PMC11695814; doi:10.1158/2767-9764.CRC-24-0471)
Supplement: Supplementary Figure S3 — TOPFLash analysis in COX-2 or EP2/EP4 inhibitor-treated AKTP R270H cells [file crc-24-0471_supplementary_figure_s3_suppsf3.pdf]

Supplementary Figure S3

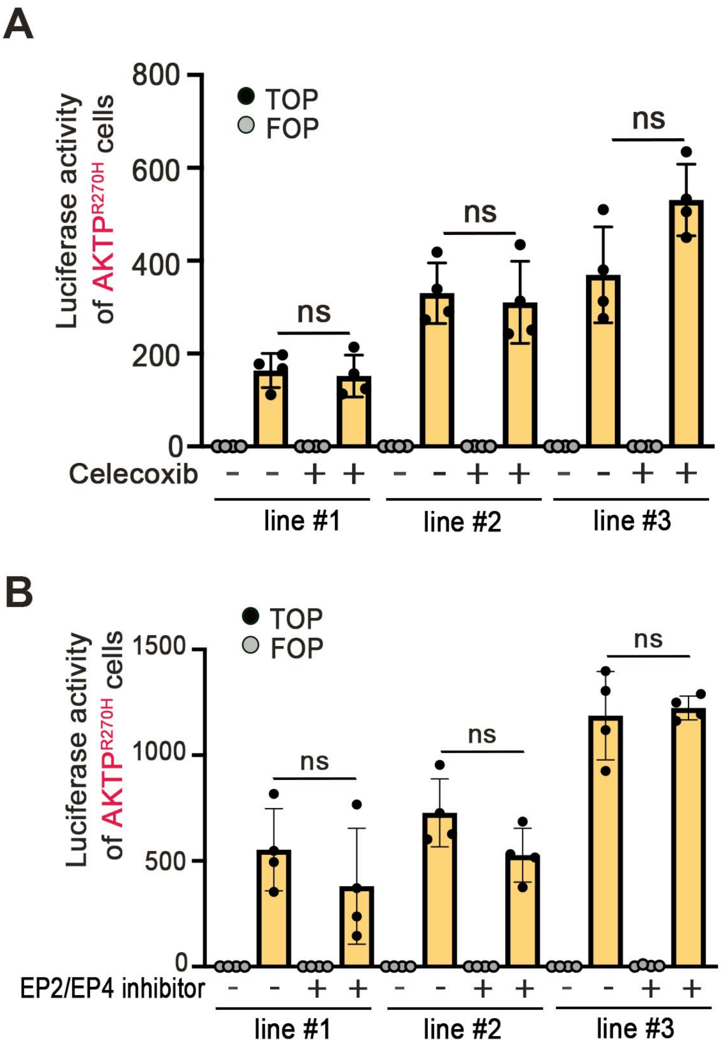

Supplementary Figure S3

COX-2/PGE$_2$ pathway-independent Wnt/β-catenin activation in AKTP^{R270H} cells. Luciferase activities of Wnt reporter assays (TOPFLASH) in AKTP^{R270H} cells in the presence or absence of COX-2 inhibitor, celecoxib (A) or EP2/EP4 inhibitors (B) are shown as bar graphs. Note that Wnt/β-catenin signaling activity is constitutively activated in three independently established AKTP^{R270H} lines, and the level of Wnt activation was not affected by inhibition of the COX-2/PGE$_2$ pathway.
